# Usability of an Embodied CAVE System for Spatial Navigation Training in Mild Cognitive Impairment

**DOI:** 10.3390/jcm12051949

**Published:** 2023-03-01

**Authors:** Cosimo Tuena, Silvia Serino, Chiara Stramba-Badiale, Elisa Pedroli, Karine Marie Goulene, Marco Stramba-Badiale, Giuseppe Riva

**Affiliations:** 1 Applied Technology for Neuro-Psychology Lab, IRCCS Istituto Auxologico Italiano, Via Magnasco 2, 20149 Milan, Italy; 2 Department of Psychology, Università Cattolica del Sacro Cuore, Largo Gemelli, 1, 20121 Milan, Italy; 3 Faculty of Psychology, Università eCampus, Via Isimbardi 10, 22060 Novedrate, Italy; 4 Department of Geriatrics and Cardiovascular Medicine, IRCCS Istituto Auxologico Italiano, Via Mosè Bianchi 90, 20149 Milan, Italy; 5 Humane Technology Lab, Università Cattolica del Sacro Cuore, Largo Gemelli, 1, 20121 Milan, Italy

**Keywords:** virtual reality, aging, dementia, spatial memory, embodiment

## Abstract

Individuals with mild cognitive impairment (MCI) usually report navigation and spatial memory impairments. Spatial navigation is an embodied process that requires the active involvement of both physical (e.g., motor commands and proprioception) and cognitive (e.g., decision-making and mental rotation) information. Immersive virtual reality (IVR) is a valuable tool that employs this information as real-world navigation does. Given the crucial impact of spatial navigation on daily life, research should focus on ways to enhance it. Though they are still in their development, contemporary IVR methods for spatial navigation training in MCI seem promising. In this usability study, eight patients with MCI syndrome tested an IVR spatial navigation training demo and interacted with the CAVE using active stereo glasses, a foot-motion pad, and a joypad. During the demo, users were asked to report their impressions on the IVR training using the thinking-aloud procedure. Moreover, questionnaires regarding usability, presence and cybersickness were administered at the end of the experience. Our results show that the first version of this system is usable by the patients even if most of them did not have experience with PC/IVR. The system provided a moderate sense of spatial presence and limited negative effects. Issues found during the thinking-aloud procedure concerned the visual aspects, which affected the interaction user-system. Participants reported that they needed more practice with the foot-motion pad even though the overall experience was positively evaluated. Identifying these critical features was essential to develop an improved version of the current system.

## 1. Introduction

In the last decades, great effort was dedicated to the design and testing of non-pharmacological treatments with innovative technologies to delay cognitive deterioration in aging. Mild cognitive impairment (MCI) syndrome has always been considered a transitional stage between normal aging and dementia; nevertheless, a proportion of MCI individuals could remain stable, or even revert to normal cognition [1,2]. Indeed, since no pharmacological therapy has been approved for MCI thus far, it can be considered as a preferred time window for non-pharmacological treatments [1]. Typically, MCI individuals with an impairment in the memory domain (amnestic MCI) are associated with a higher risk of developing Alzheimer’s disease (AD), conversely, individuals with non-amnestic MCI (i.e., MCI that affects other cognitive domains than memory) can progress to other neurodegenerative diseases such as vascular, frontotemporal, or Lewy bodies dementias [3]. Among the cognitive markers of this stage, navigation, and spatial memory deficits have often been overlooked but are nowadays increasingly being studied. The inability to find one’s way through an environment, to remember item/place locations, or to learn new paths can be considered crucial markers for AD diagnosis [4]. Importantly, these impairments usually occur before dementia onset and are found to be already observable in MCI with amnestic and non-amnestic subtypes [5]. Recent studies have underlined that navigation and spatial memory impairments can also be observed in other neurodegenerative diseases and dementias [6,7,8,9]. Hence, the training of spatial navigation in MCI aims at training and improving cognitive skills and brain regions involved in this function [10].

Spatial navigation refers to the ability to estimate one’s position employing both environmental (e.g., landmarks, boundaries) and bodily cues (e.g., sensorimotor system, proprioception, vestibular information) [11] and spatial memory supports the ability to learn, store, and recall paths and locations [12]. The spatial information can be organized according to two different frames of reference: the egocentric (body-centered) and the allocentric (world-centered) representations [13]. In other words, we can remember a position of an item by using our body (e.g., “the church is to my right”) or by using item relations (e.g., “the church is close to the mall”). In addition to these spatial cognitive processes, other cognitive domains including spatial attention (e.g., focusing attentional resources on landmarks), spatial information manipulation (e.g., using maps), and decision-making (e.g., route planning) are involved [14]. Importantly, a recent study suggested that navigation and spatial memory can be considered as embodied processes where an abstract cognitive representation of the space is supported by action, perception, and bodily information [10]. Therefore, bodily information and the interaction with the environment provide useful information to develop cognitive maps of the space and recall this information. Indeed, a recent study concerning the embodiment mechanisms of aging and neurodegenerative diseases suggested that the sensorimotor system’s deterioration contributes to spatial impairments [15].

Immersive virtual reality (IVR) represents a suitable tool for navigation and spatial memory training as it enhances the manipulation of bodily and environmental information [10]. IVR enables the creation of multisensory experiences close to those in the real world where the user can interact with the body and the environment [16]. Therefore, IVR can be considered an embodied technology [17]. A recent systematic review underlined that the inclusion of sensorimotor cues and route decision-making during virtual navigation tasks enhances spatial memory [18]. According to a recent study, active virtual navigation through sensorimotor information can improve allocentric spatial memory in MCI compared to passive navigation (i.e., viewing the navigation on a screen) [19]. Similarly, directional cues and salient landmarks have been proven to be helpful for spatial navigation and memory tasks in patients with AD and MCI [20].

The assessment of the usability and acceptability of IVR systems is a critical step that anticipates clinical trials and pilot studies. According to Tuena and colleagues [21], it is crucial to carefully consider the following factors: (1) to identify barriers and facilitators of a specific population for a given virtual reality technology; (2) to design the task centered on the target population; and (3) to employ mixed methods for the assessment of the usability and IVR-related aspects. Recent usability studies have shown that IVR is rated as an acceptable, usable, and tolerable system for cognitive training by older people with MCI. Nevertheless, cybersickness could be one of the factors that might affect the acceptance and ease of use of an IVR system in MCI [22]. Cybersickness can include a series of symptoms arising during the experience in IVR, which may include nausea, vision discomfort, headache, dizziness, and disorientation [22]. A usability study has also been proven to identify those elements that could prevent the use of the technology [23,24]. Indeed, it is crucial to ensure that technologies are available and can be used by older individuals with cognitive impairment [25]. Assessing the usability and user experience of cognitive intervention employing new technologies for people with MCI or dementia offers a comprehensive perspective that can help with the development of these technologies following the requirements and characteristics of the target population [26].

This study aimed to assess, with both qualitative and quantitative methods, the usability, side effects, and immersion of an embodied-based CAVE (Cave Automatic Virtual Environment) in patients with MCI. Currently, few CAVE interventions for cognitive/motor training have been proposed (e.g., [27,28]), and further studies are required to test the efficacy of embodied CAVE spatial training. The embodied-based CAVE system was developed in the ‘Active Navigation Training: an innovative embodied-based training system for spatial navigation in aging’ project (acronym: ANTaging). Based on these premises, ANTaging was designed.

## 2. Materials and Methods

### 2.1. Participants

Eight (two females and six males) patients with MCI syndrome were recruited for the usability study. The mean age was 72.75 (SD = 5.56), the mean year of education was 9.63 (SD = 3.77), and the mean of the mini-mental state examination (MMSE) was 25.85 (SD = 1.75). MCI diagnosis was carried out by a clinical neuropsychologist and by the physician (CSB, EP, KMG), according to the patient history, neurological referral, and neuropsychological diagnosis. MCI diagnosis was carried out according to the core clinical criteria of Albert and colleagues [29]: (1) concern regarding a change in cognition obtained from the patient, an informant, or a clinician; (2) impairment in one or more cognitive domains (as assessed by clinical neuropsychologist cognitive tests); (3) preservation of independence in functional abilities; (4) and no dementia diagnosis (as reported by patient history and anamnesis). Additional inclusion criteria were: the absence of severe cognitive deterioration as assessed by the Italian version of the MMSE [30] and age ≥ 60. Exclusion criteria were: (i) the presence of acute stroke/transient ischemic attack; (ii) the presence of other concomitants severe neurological/psychiatric diseases; (iii) history of traumatic brain injury with loss of consciousness; (iv) physical/functional deficits that could affect the use of IVR; (v) severe visual deficiency; and (vi) the presence of recurrent vertigo. A sample size between five and ten was considered adequate [21].

Participants were recruited at the Outpatient Clinic of the Department of Geriatrics and Cardiovascular Medicine, IRCCS Istituto Auxologico Italiano—Mosè Bianchi, Milan. The study was approved by the Ethics Committee of Istituto Auxologico Italiano and written informed consent was obtained from the participants before they participated in the study.

### 2.2. Equipment

CAVE is a 3D immersive four-walled virtual room in which the 3D visualization of the virtual environments occurs through the combination of four stereoscopic projectors (Full HD 3D UXGA DLP), three rear-projection screens (i.e., the three walls), and one downward-projection screen, all having a projectable area of 266 × 200 cm. A cluster system composed of two HPZ620 Graphics Workstations, mounting an Nvidia Quadro K6000 GPU with dedicated Quadro Sync cards, is responsible for the rendering of the four projection surfaces, user tracking, and functional logic. CAVE is equipped with a Vicon motion tracking system, with four infrared cameras with 1-megapixel resolution, which allows for the tracking of specific reflective markers positioned on target objects and the correct reading of the simulated spaces and distances with a 1:1 scale ratio, thus enhancing the feeling of being immersed in the virtual scene. For this study, a 3dRudder (https://www.3drudder.com/) and an Xbox controller were used to interact with the virtual environment. The 3dRudder is a circular platform that is used while sitting in a comfortable and safe position. The patient places the feet on the top to use it. It is, in turn, fixed on a semi-spheric lower section. This solution allows the user to manage the movement intuitively, tilting the feet in the desired direction. Inside the device are installed inertial sensors and pressure sensors that process the user’s movements and translate them into virtual actions. Furthermore, Vicon markers are used to track the position of the 3D glasses and the head direction of the participant. Four speakers are present at each angle of the CAVE to provide immersive audio. The virtual environment was designed and implemented using Unity software and displayed in the CAVE using MiddleVR.

### 2.3. Protocol

The usability session lasted approximately 30 min and the participants were asked to complete a short ANTaging demo training in the CAVE. As described in the ANTaging protocol [31], the demo session was composed of an encoding phase followed by a recall phase.

Before starting the task, the participants received training on how to utilize the 3dRudder. Afterward, according to the thinking-aloud procedure (see Section 2.4), they were asked to express their thoughts and comments in detail during the experience of the demo. The environment was a circular city square, in which different landmarks were present: an obelisk, a distant mountain range, the clouds, and an arcade that surrounded the square. At the beginning of the task, participants were asked to turn around, using the 3dRudder, to look for some attentional cues (orange spheres appeared at the top of the screen next to which a number from 01 to 06 was written), and they were asked to carefully observe the environment. At the top of the frontal wall, the following sentence appeared, ‘find attentional cues’. Once the spheres were found, participants had to use the joypad (button ‘A’) to make the attentional cues disappear and let the directional cues come out (a line to follow). At this point, another message appeared on the frontal screen of ‘find the guideline’. They had to use this line to reach one object and learn its location. This part was repeated four times. On the screen, they could also see an interactive map with cardinal points indicating their position, the object, and the locations of the landmarks. After this encoding demo, the users tried the recall demo phase. They had to recall the object’s exact location and to collocate the object by pressing ‘A’ on the joypad when they were convinced. As a result, a message was shown: ‘congratulations’ if the position was right (within six virtual units from the actual location) or ‘try again’ if it was wrong. This procedure was carried out four times and the obelisk or the arcade was randomly presented to force the use of egocentric or allocentric recall strategies [31]. In the recall phase, none of the encoding cues (attentional cues, guidelines, and map) were provided. The only recall cue was a white circle on the ground that indicated the right position (made it appear by the experimenter through the keyboard). In the fourth recall trial, participants were asked to look for this marker and go to its location. Relaxing music was used as a background sound during the demo. At the end of the demo, they were given some questionnaires to complete aimed at evaluating their user experience.

### 2.4. Measures

The experience in the CAVE was evaluated using the following methods.

The system usability scale (SUS) [32] is a “quick and easy to use” questionnaire composed of 10 items in which users need to express the degree of agreement on a 5-point Likert scale, from ‘strongly disagree’ to ‘strongly agree’ for each statement. SUS has proven to be a valuable evaluation tool, being robust and reliable to evaluate a wide range of technologies [32]. The final score can range from 0 ‘lack of usability’ to 100 ‘optimal usability’. Scores were interpreted according to the 7-point adjective rating scale [33], which is composed of the following levels: ‘best imaginable’, ‘excellent’, ‘good’, ‘OK’, ‘poor’, ‘awful’, and ‘worst imaginable’.

The Independent Television Commission—Sense of Presence Inventory (ITC-SOPI) [34] is a 44-item, self-report questionnaire that investigates several aspects of the IVR experience. Participants are required to rate their degree of agreement–disagreement with a 5-point Likert scale from 1 ‘strongly disagree’ to ‘strongly agree’. The scoring is obtained by calculating the mean of all completed items contributing to each factor. Specifically, it measures the sense of physical space (SOPS), engagement, ecological validity, and the negative effects of the VR experience. We administered only two subscales: the SOPS and the negative effects.

Thinking-aloud [35] is a qualitative method that is generally administered to assess usability when a new technology is developed. Users are asked to express their opinion regarding the technology use and criticism while interacting with the device/software during the task [35]. The experimenter was asked to take notes or to record the participants’ observations. All the verbalizations were transcribed into a reporting grid and analyzed with thematic analysis to develop the formal usability report. The grid was composed of interactive tasks that represent different interactions with the environment and the main actions that the user had to do. The tasks are explained below.

- Does the user read attentional cues well?: At the top of the screen, the following sentence appears ‘find attentional cues’.
- Attentional cues search: Indicate if the user can find the orange spheres that appear around the arena.
- Does the user use the button to make them disappear?: The user has to use the joypad (the button ‘A’) to make attentional cues disappear.
- Does the user read the sentence well?: A message appears ‘find the guideline’.
- Guideline search: Indicate if the user uses the line to reach the object.
- 3dRudder rotation to find the object: Indicate if the user turns the 3dRudder correctly to find the object.
- Does the user see the object well?: Indicate whether the user sees the object well.
- Advancement with the 3dRudder to reach the object: Indicate if the user easily uses the 3dRudder to reach and pick the object.
- Does the user read the 3D map well?: On the screen, the user can see a map with cardinal points indicating their direction and the landmarks’ locations.
- Object relocation: The user has to relocate the object four times using the 3dRudder to reach the correct point.
- Does the user use the button to relocate?: The user has to use the joypad (button ‘A’) to put the object in the preferred location.
- Does the user read the relocation feedback well?: A message is shown on the screen ‘congratulations’ if the position is right or ‘try again’ if it is wrong.
- Does the user see the position marker well?: The user has to go to the exact location of the item looking for a white circle on the ground that indicates the correct location.

In addition, right after the demo, users were asked (“How did you find this system?”) to express any issues they found during the interaction, and the responses were analyzed with thematic analysis.

## 3. Results

### 3.1. Quantitative Measures

**Figure 1:**
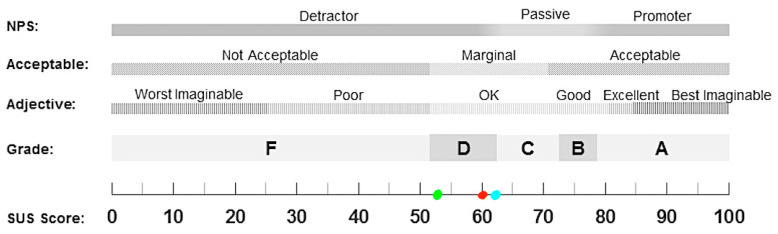
Usability of the ANTaging system; red bullet point = total mean score; green bullet point = females mean score; light blue bullet point = males mean score. SUS = System Usability Scale.

Most of the participants reported that they did not have computer experience (37.5%) or that they had sufficient (37.5%) computer experience. Almost all of the participants (87.5%) did not use video games. Some individuals (37.5%) had already used an IVR system, while others (62.5%) did not. Most of them (62.5%) did not know IVR before this demo session. The mean score of SUS was 60 (SD = 15.05). According to Bangor and colleagues [33], this score places ANTaging in a marginal zone between high and low acceptability and the level of usability can be defined as “OK,” as shown in Figure 1. In particular, among women, the mean was 53.75 and among men, it was 62.08. Therefore, men rated that the system had better usability than women. Despite the predominant unfamiliarity of patients with technology, this prototype version was found to be usable. Among the users who had already used an IVR system, one person (12.5%) obtained a ‘poor’ score in SUS, one person (12.5%) obtained an ‘OK’ score, and another one (12.5%) obtained an ‘excellent’ score. Instead, among the users who had never used an IVR system, two users (25%) obtained a ‘poor’ score in SUS, one person (12,5%) obtained an ‘OK’ score, one person (12.5%) obtained a ‘good’ score, and another one (12.5%) obtained an ‘excellent’ score. This indicates heterogenous usability according to previous IVR experience, although 25% of the patients who never tried IVR rated the system as ‘poor’. The mean score of cybersickness was 1.23 (SD = 0.31), indicating that the users did not have any kind of negative effects. Finally, the mean score of spatial presence was 2.93 (SD = 0.75), showing above average (i.e., 2.5) sense of presence in the virtual city square. The results are presented in Table 1.

### 3.2. Qualitative Measures

When interacting with the ANTaging system, users expressed some concerns regarding the ‘visual’ (theme) features, which had a significant impact on the higher-order category ‘interaction’. Users experienced some difficulties in seeing the object shown on the screen. The task ‘Does the user see the object well?’ was not completed by 25% of the individuals.

*“I don’t see any objects”*.

(ID 3)

They also reported some issues in understanding the map. Indeed, the task ‘Does the user read the 3D map well?’ was not concluded by 25% of individuals.

*“I’m towards the Northwest, I don’t read well”*.

(ID 1)

Furthermore, the patients expressed some difficulties in reading the feedback on their actions from the system. They said that the sentence disappeared too quickly, not providing them with enough time to read. A total of 100% of users failed the task ‘Does the user read the relocation feedback well?’

*“It’s too fast! I didn’t read it in time!”*.

(ID 1)

Feedback after the use of the system highlighted other two themes, ‘ability’ and ‘fun’. It emerged that more practice is required when using a new device.

*“I have to do some practice”*.

(ID 2)

*“It seemed like an easy game, once you understand how you have to move there is no problem”*.

(ID 3)

*“It’s the first time I’ve used the platform and the joystick, I’m not skilled”*.

(ID 8)

Regarding the second theme, other users expressed a different opinion, indeed, most of the people stated that they liked the game.

*“Then I liked it”*.

(ID 4)

*“I liked it”*.

(ID 6)

*“I don’t really like games”*.

(ID 8)

## 4. Discussion

This study aimed at investigating the usability, the side effects, and the immersion of an embodied-based CAVE in MCI patients, through both qualitative and quantitative methods. To our knowledge, this is the first study that investigated the usability of a CAVE system for spatial memory training in MCI. The assessment of the usability and acceptability of IVR systems is a crucial aspect before clinical trials.

The qualitative part of this study consisted of collecting comments and feedback from the users and this analysis underlined that most of the participants appreciated using the ANTaging system, but also suggested some adaptations. For instance, the users reported some visual issues while completing the task. They experienced some difficulties because some objects were barely visible because of the texture, they could not completely understand the 3D map, and they could not always know if the response was correct as the feedback disappeared too quickly.

Regarding the feedback after the game, we found that the ability to use technology needs to be improved by practicing with the foot-motion pad. Furthermore, the experience, in general, was positively evaluated by users. These post-experience comments allowed us to bring out some psychological aspects, where the first theme was relative to self-efficacy and the second was to one’s feelings regarding this training.

As far as the quantitative data are concerned, the SUS analysis underlined that, despite the lack of familiarity with technology, the system was rated as usable. The ITC-SOPI scores revealed that spatial presence was moderate and very low negative effects (e.g., cybersickness) were reported. This study allowed us to identify barriers that could affect the use of the CAVE system in MCI individuals. Our results were confirmed by recent studies on the usability of an IVR cognitive training that was rated as an acceptable, usable, and tolerable system for cognitive training by patients with MCI and subjective cognitive decline [23,24]. It could be argued that IVR may be used safely by older adults suffering from MCI. Finally, our results showed that in addition to the graphic aspects, in older people with MCI, psychological factors such as the perceived self-efficacy of the use of a new technological device are of great importance. This is perfectly in line with technology acceptance models in aging [26]. The thinking-aloud protocol represents a valuable procedure to collect the users’ feedback about their experience with the technology. Older people with cognitive impairment may experience some difficulties in using technology. Based on these premises, it is crucial to understand if the technology is easy to use to achieve the therapeutic goals and whether the user perceives it as pleasant [26]. The results of our usability study concerned the visual and graphic features of the system (i.e., sizes of the objects and signs). At the same time, some patients were not confident about using the 3dRudder, however, this can be improved by practicing with the device.

Another important aspect to consider is the so-called ‘transformation of flow’. This is the ability of the patients to exploit a flow experience during IVR training and identify and use unexpected psychological resources [36]. IVR through a strong sense of presence triggers an empowerment process because it allows one to link intentions and actions in the virtual world. Therefore, fixing potential usability issues that could affect the sense of presence and immersion in the virtual training could enhance its outcomes [37]. We acknowledge that our work had some limitations. The first is related to the small sample size. However, a previous usability review confirmed that 5–10 users could express most of the issues of technology [26]. In contrast, the analysis of both qualitative instruments and questionnaires can be interpreted as a strength of our study. This usability study was carried out employing a CAVE system including four projectors projecting the virtual scenes in an area of 266 × 200 cm. A more immersive alternative consisted of a room of six faces. 3dRudder enables the experimenter to safely process the users’ motor commands and proprioceptive information of the lower limbs while performing spatial navigational tasks. Nevertheless, a limitation of this device is that it does not involve whole-body proprioceptive and vestibular information. Finally, it might be interesting to assess usability after the sessions of IVR training to investigate whether the experience with the technology improves and becomes easier to use. The mean score of SUS was 60, which cannot be considered as an excellent result. Nevertheless, this usability study is a pre-test training that was carried out on a prototype of the application, (i.e., the first version of the system). We aim to improve the most critical features of the system before starting the clinical trial to advance the research in the field. Additionally, after having demonstrated the efficacy and effectiveness of the proposed system, a cost–benefit analysis could be a future step of the ANTaging project.

## 5. Conclusions

This study aimed at investigating the acceptance and usability of an IVR system in older adults with MCI. It is crucial to provide spatial navigation training to this population since studies have shown that, independent of the underlying etiology or MCI phenotype, both subjective and objective spatial memory impairments are present [5]. Additionally, navigation is an essential daily-life ability, thus preserving it intact is essential to the independence of MCI patients. The results of the present study demonstrated that the ANTaging system can be used by patients who suffer from MCI, even if they have no experience with this device. Usability is a crucial step for clinical research in aging as it enables one to identify potential issues that could affect cognitive training adherence, effects, and pleasantness.

## Figures and Tables

**Table 1:** Usability characteristics.

Item	Scores
PC experience	
None (N)	3
Sufficient (N)	3
Good (N)	1
Great (N)	1
VG experience	
Never (N)	7
Occasionally (N)	1
Often (less than 50% of the days) (N)	0
More than 50% of the days (N)	0
Everyday (N)	0
VR experience	
Yes (N)	3
No (N)	5
VR knowledge	
None (N)	5
Sufficient (N)	2
Good (N)	1
Great (N)	0
SUS	60 (SD = 15.05)
Females	53.75 (SD = 8.75)
Males	62.08 (SD = 16.10)
Cybersickness (ITC-SOPI)	1.23 (SD = 0.31)
Spatial presence (ITC-SOPI)	2.93 (SD = 0.75)

VG = video game; VR = virtual reality; SUS = System Usability Scale; ITC-SOPI = Independent Television Commission—Sense of Presence Inventory.

## Data Availability

Data are available upon request.
